# Reliability and Agreement of Automated Head Measurements From 3-Dimensional Photogrammetry in Young Children

**DOI:** 10.1097/SCS.0000000000009448

**Published:** 2023-06-12

**Authors:** Tareq Abdel-Alim, Pauline Tio, Melissa Kurniawan, Irene Mathijssen, Clemens Dirven, Wiro Niessen, Gennady Roshchupkin, Marie-Lise van Veelen

**Affiliations:** * Department of Neurosurgery, Erasmus University Medical Center; † Department of Radiology and Nuclear Medicine, Erasmus University Medical Center; ‡ Department of Plastic and Reconstructive Surgery, Erasmus University Medical Center, Rotterdam; § Faculty of Medical Sciences, University Groningen, Groningen, The Netherlands; ∥ Department of Epidemiology, Erasmus University Medical Center; ¶ Child Brain Center, Erasmus MC Sophia Children's Hospital, Rotterdam, The Netherlands

**Keywords:** Anthropometrics, automated measurements, craniosynostosis, 3D photogrammetry, head measurements

## Abstract

This study aimed to assess the reliability and agreement of automated head measurements using 3-dimensional (3D) photogrammetry in young children. Specifically, the study evaluated the agreement between manual and automated occipitofrontal circumference (OFC) measurements (n = 264) obtained from 3D images of 188 patients diagnosed with sagittal synostosis using a novel automated method proposed in this study. In addition, the study aimed to determine the interrater and intrarater reliability of the automatically extracted OFC, cephalic index, and volume. The results of the study showed that the automated OFC measurements had an excellent agreement with manual measurements, with a very strong regression score (*R*^2^ = 0.969) and a small mean difference of −0.1 cm (−0.2%). The limits of agreement ranged from −0.93 to 0.74 cm, falling within the reported limits of agreement for manual OFC measurements. High interrater and intrarater reliability of OFC, cephalic index, and volume measurements were also demonstrated. The proposed method for automated OFC measurements was found to be a reliable alternative to manual measurements, which may be particularly beneficial in young children who undergo 3D imaging in craniofacial centers as part of their treatment protocol and in research settings that require a reproducible and transparent pipeline for anthropometric measurements. The method has been incorporated into CraniumPy, an open-source tool for 3D image visualization, registration, and optimization, which is publicly available on GitHub (https://github.com/T-AbdelAlim/CraniumPy).

The measurement of head circumference, also referred to as the largest occipitofrontal circumference (OFC), has been an essential part of the physical examination of children for decades.^1^ It is a meaningful parameter for brain development and cranial growth and serves as an important indicator for abnormal cranial development in young children (eg, due to malnutrition or (congenital) craniofacial anomalies).^2–4^ The OFC is also a significant predictor of intracranial volume (ICV), another important cranial growth parameter.^5^ However, a major drawback is that it usually requires conventional imaging methods, such as computed tomography (CT) or magnetic resonance imaging (MRI), which are not preferred during follow-up due to the required sedation (MRI) and the possible effects of radiation (CT) in pediatric patients.^6^

These growth parameters are often combined with the Cephalic Index (CI), a parameter that describes the axial shape of the head as a ratio between the maximum head width and length.

Although conventional measurements, such as the OFC and CI are unable to fully convey the complex development of the skull in 3 dimensions, they are useful, simple, and inexpensive measurements that can be obtained manually and will likely stay deeply rooted in clinical practice.^7^ However, manual measurements are prone to human error, and acceptability, in especially young children, can be limited.^8^ Although protocols usually state how these manual measurements should be obtained, finding the most posterior part of the skull, temporal regions, and the most anterior part of the forehead, while keeping the measuring tape horizontal and in direct contact with the skin, is largely influenced by the experience and perception of the practitioner.

Nowadays, anthropometric measurements that are obtained from digital 3-dimensional (3D) models are frequently utilized and accepted.^9–13^ Accurate 3D models of the patients’ surface can be reconstructed from CT scans or acquired using 3D photogrammetry. The latter has shown to be an accurate and reliable alternative when no information about intracranial structures is required.^14–16^ Three-dimensional photogrammetry uses optical sensors, making it radiation and sedation-free and less stressful for children compared with manual measurements, while it allows quantitative shape analysis that extends beyond conventional anthropometric measurements.^8,17^

Accurate and quantitative head measurements are crucial for pediatric patients with craniofacial conditions, such as craniosynostosis, which can cause craniofacial dysmorphologies due to the premature closure of one or more cranial sutures. Deviations in head measurements, particularly OFC, can indicate postoperative relapse or be an early sign of raised intracranial pressure and require further investigation.^5,18–20^ Cranial growth is monitored during regular follow-up moments as it may be impaired due to the nature of the condition, or as a result of surgical intervention. Excessive OFC values, in contrast, are associated with hydrocephalus.^19^

In addition to conventional (manual) measurements and checkups, our patients undergo regular 3D photogrammetry images to evaluate the cranial shape development over time.

Several studies focused on the use of head measurements based on 3D imaging, which has shown to be a reliable modality with comparable outcomes to manual measurements.^13,21–23^

To reduce the number of physical measurements, and increase the accuracy and reproducibility of head measurements in our pediatric craniofacial patients, we have developed an algorithm that can be used to automatically extract the OFC and other head measurements (CI and volume) from a 3D image.

The aim of this study is to evaluate the agreement between the reliability of these automatically and manually obtained OFC measurements and to determine whether these can be used interchangeably. In addition, the interrater and intrarater reliability of the OFC, CI, and mesh volume (above the nasion-tragus plane) is determined using a random subset of 3D images.

## METHODS

### Study Sample

Three-dimensional images were retrospectively collected from patients up to the age of 6 with sagittal synostosis who were treated in our craniofacial center between 2000 and 2019. Patients from this particular cohort were selected for this validation study as they all underwent 3D imaging and regular cephalometric measurements during follow-up, as dictated by the treatment protocol.^19^

The agreement between automatically and manually extracted OFC measurements is evaluated over the entire included data set. A random subset is used to determine the interrater and intrarater reliability of the automatically extracted OFC, CI, and volume.

Three-dimensional images were acquired using a 3dMDhead system and were included if the patient had a manual OFC measurement within 4 days of image acquisition to exclude natural growth as a source of error in this study. Images in this set were assessed on image quality to ensure that included images were free from imaging artifacts, protruding hair, or postoperative swelling. The study protocol was approved by the Institution’s Medical Ethical Committee (MEC-2016-312) and followed the statements of the Declaration of Helsinki.

### Manual Occipitofrontal Circumference Measurements

A retrospective collection of manually acquired OFC measurements from patient records was performed. Manual OFC was obtained using a standardized measuring tape by a skilled clinician in accordance with the hospital protocol. According to the protocol, the measuring tape has to include the most posterior part of the skull, the temporal region, and the forehead. In addition, the measuring tape has to be horizontal, above the ears, and in direct contact with the skin.

### Image Processing and Extraction of Occipitofrontal Circumference and Cephalic Index Measurements

**FIGURE 1:**
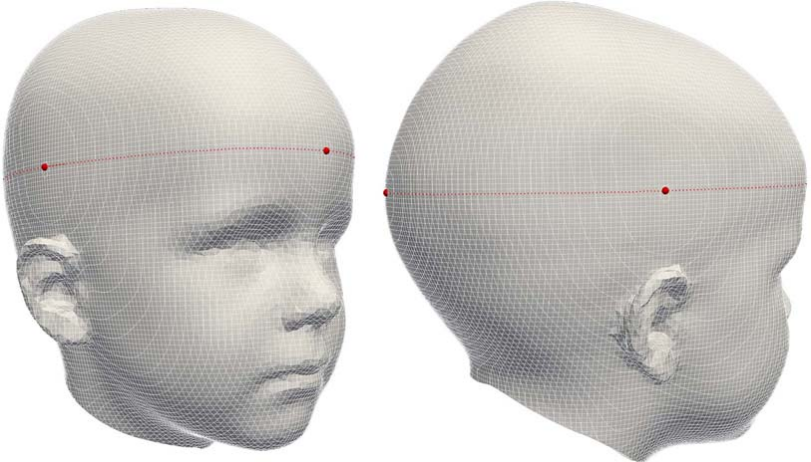
Contour lines (white), local optima (red dots), and extracted OFC (red line). OFC indicates occipitofrontal circumference.

The algorithm that was developed to extract the head measurements assumes the 3D image to be in a predefined reference frame. Therefore, aligning the 3D image data to a standard coordinate system that is consistent across different images is a crucial step. Details about the registration steps are provided in Supplemental Methods: Image Processing (Supplemental Digital Content 1, http://links.lww.com/SCS/F114) Although finding an anatomically meaningful reference point based on surface data is not trivial, multiple reference frames have been defined in the literature.^13,14,23–26^ The method presented in this study uses the plane going through the nasion and both tragi as an alternative to the commonly used orbitomeatal line, as the tragus is often easier to identify on 3D images than the outer canthus (particularly young in children). These landmarks are selected by the user and guide the global (initial) alignment. After alignment, an iterative search algorithm goes systematically through different axes in pursuit of local optima. The maximum head length is extracted by searching along axial slices ( verticalspacing=1mm ). These slices are a function of 2 variables (coordinates) along a particular axis and can, therefore, be mapped as contour lines on top of the 3D image. These contour lines provide information about the steepness of slopes in a similar way to contour lines on topographic maps, which connect points of equal elevation. For our application, contour lines plotted along the *y*-axis [posterior (−) to anterior (+)] converge at 2 locations: one on the anterior and one on the posterior part of the head. The distance between these 2 optima is defined as the maximum head length. To improve the reproducibility of this method, searches are limited to orthogonal slices, meaning that the anterior and posterior optima points have to lie within a single plane that is parallel to the xy-plane defined in our reference frame, that is, any plane parallel to the plane going through the nasion and both tragi. The slice containing the maximum head length, referred to as the occipitofrontal (OFD) plane, is subsequently used to find the head width by searching for 2 optimum points on the parietal sides of the head. Vertical slices parallel to the yz-plane [left (−) to right (+)] converge at these points. The contour lines (in white) and optima (as red dots) are visualized in Figure 1.

**FIGURE 2:**
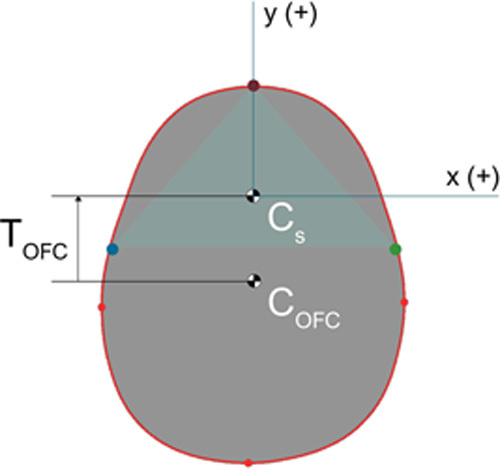
Center-of-mass translation. OFC indicates occipitofrontal circumference.

No landmark used for the global alignment (nasion and both tragi) is located on the posterior part of the head, which makes any follow-up shape analysis less sensitive to posterior changes. Therefore, the choice was made to include a final center-of-mass–based translation that translates the 3D image from the initial anchor point (the centroid of the 3 landmarks) to a new anchor point $C_{\mathrm{OFC}}$ that is a function of all regions of the head (Fig. 2). The red slice going through the 4 optima is a set of xy-coordinates (within the same *z*-plane). The final 3D image translation $T_{\mathrm{OFC}}$ is found by first calculating the centroid $C_{\mathrm{OFC}}$ of the red slice and then applying a translation that is equal to the difference between $C_{\mathrm{OFC}}$ and the initial anchor point $C_{S}$ . As by definition $C_{s}=\;(0,0,0)$ , the translation is obtained: $T_{\mathrm{OFC}}\;=\;-C_{\mathrm{OFC}}$ .

This is a single-axis translation (along the *y*-axis in the anterior-posterior) and preserves asymmetries (if present) between the left and right-hand side of the head during follow-up analysis.

**FIGURE 3:**
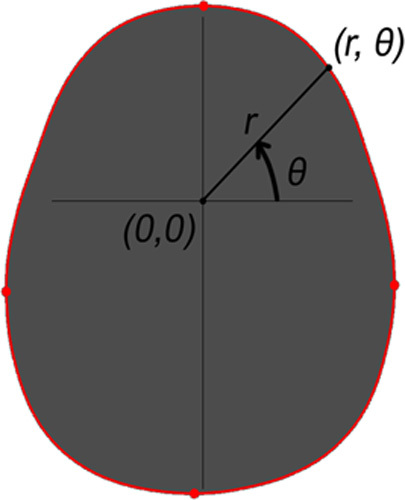
OFC from axial slices using polar coordinates. OFC indicates occipitofrontal circumference.

To obtain the OFC, the set of cartesian coordinates that make up the red slice in Figure 3 is converted into polar coordinates (Eq. 1) and sorted $\left[\theta_{\min};\theta_{\max}\right]$ . The OFC is then approximated by taking the sum of the distances between consecutive coordinates (Eq. 2), beginning at θ=0 until θ=2π(radians) for all n vertices that make up the line.

Eq. 1 $$\left(r_{i},\;\theta_{i}\right)\;=\;\left(\sqrt{xi^{2}+yi^{2}},\;\frac{y_{i}}{x_{i}}\right)$$

Eq. 2 $$\mathrm{OFC}\;=\;\sum_{i}^{n}\sqrt{{r_{i}}^{2}+{r_{i+1}}^{2}+1^{2}-r_{i}r_{i+1}\cos\;\left(\theta_{i}-\theta_{i+1}\right)}$$

### Volume Measurement

To acquire a useful mesh volume that could be used to approximate the ICV, 3D images are clipped through the annotated landmarks. This plane, near-identical to the plane going through the lateral canthus and the tragus, shows a strong correlation with ICV values from CT, but consistently overestimates the actual ICV value by ~35% to 45%, for which a correction factor needs to be applied, depending on the study population.^26,27^

In this study, we determine the interrater and intrarater influence on the extracted uncorrected mesh volume.

### Statistical Analyses: Agreement, Bias, and Reproducibility

The OFC measurement agreement between measurements from 3D images using the automated algorithm and manual measurements using a tape measure was determined for the entire data set and benchmarked against limits of agreement (LoA) for manual head measurements.^28^ Measurement agreement was expressed as the mean difference (MD), LoA, and SE. Bland-Altman plots were used to visualize the agreement between the two methods. In these plots, the *x*-axis represents the mean of the manual and automated measurement, whereas the *y*-axis contains the difference between the two.

Interrater and intrarater reliability were determined for the automatically extracted OFC, CI, and volume, based on a subset of 3D images, which were annotated twice by 2 annotators. The 3 landmarks required for registration were annotated 1 week apart to prevent memory bias. However, no measurement agreement with respect to a ground truth was determined for the CI and ICV in this study. The main concerns were incomparable modalities (CI from plain radiography) and lack of reference data (ICV from CT/MRI).

Python statistical library Scipy was used for statistical analysis.^29^ After the assumptions of normality (Shapiro-Wilk test) were confirmed, the paired *t* test was used to determine whether the manual OFC measurements differed significantly from the automatically extracted OFC values. A *P* value <0.05 was considered statistically significant and a regression score ${(R}^{2})$ was calculated.

## RESULTS

### Study Sample

To evaluate the agreement between the automatically and manually obtained OFC measurements, a total of two hundred sixty-four 3D images from 188 scaphocephaly patients (85% males and 15% females) were included in this validation study. Patients had a mean age of 37 (±21) months at the time of image acquisition and manual OFC measurement, with a mean OFC of 50.9 cm and 51.0 cm in the manual and 3D groups, respectively.

The intrarater and interrater reliability of the OFC, CI, and volume were evaluated for 2 raters who independently annotated a subset of 50 randomly selected 3D images twice. The patients in this subgroup had a mean age of 41.8 months with a mean OFC of 51.4 cm and a CI of 74.2%. The mean mesh volume was 2104.3 cm^3^. This is the volume above the nasion-tragus plane, which is a commonly extracted volume before correction based on CT correlations to approximate the volume of the cranial cavity as outlined by the cerebral contours.^16^ Mean values and SDs of the 3 extracted head measurements from this subset are also presented in Supplemental Table (Supplemental Digital Content, Table 1, http://links.lww.com/SCS/F115).

### Measurement Agreement

**FIGURE 4:**
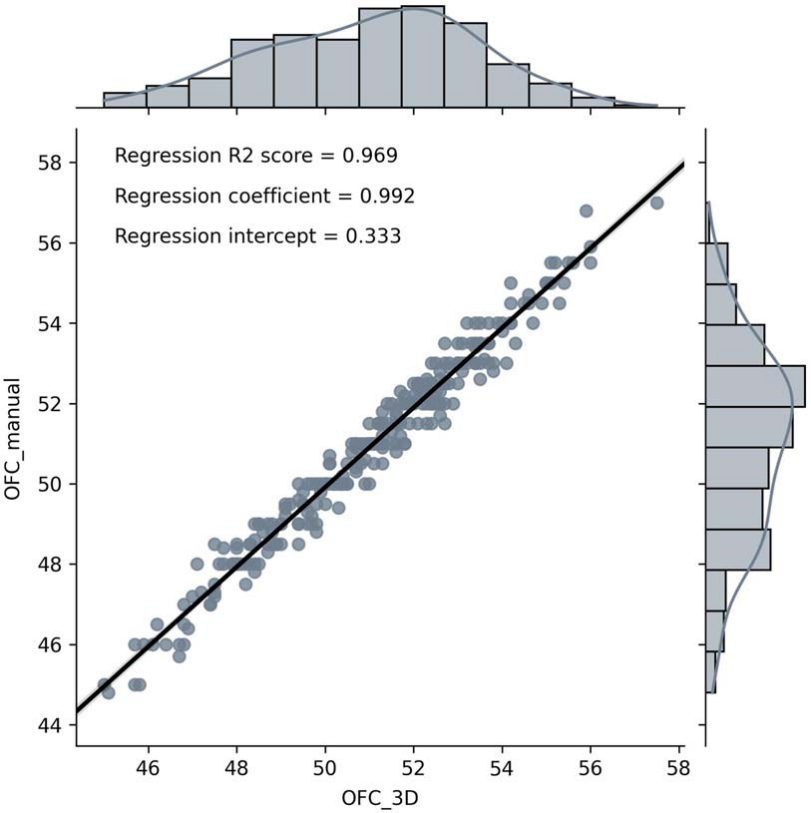
Regression analysis OFC measurements (n = 264). OFC indicates occipitofrontal circumference.

**FIGURE 5:**
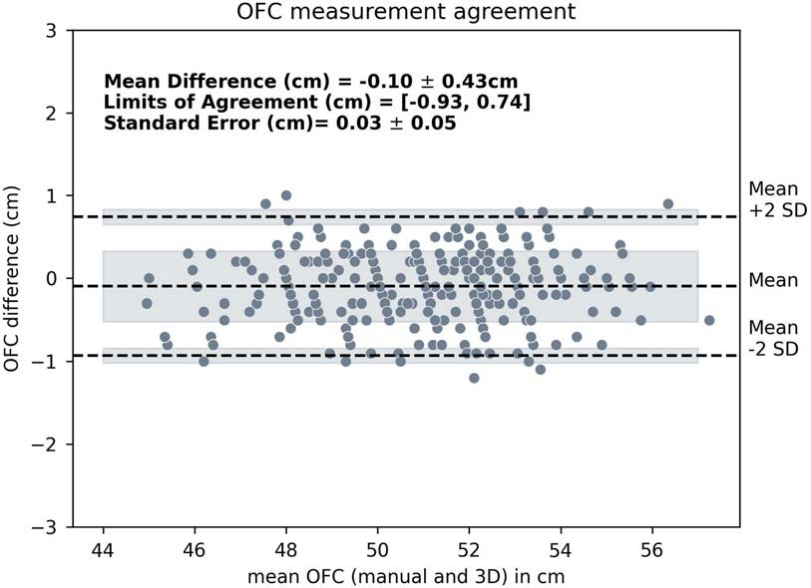
Measurement agreement OFC measurements (n = 264). OFC indicates occipitofrontal circumference.

A high regression score (*R*^2^ = 0.969) was found between the manual and automated OFC methods after comparing 264 pairs of OFC measurements (Fig. 4). After normality was confirmed, a paired *t* test showed no significant difference (*P* = 0.641) between the two sets of OFC measurements. Excellent agreement was found with a MD of −0.1 cm (−0.2%), LoA ranging from −0.93 to 0.74 cm, and a SE of 0.03 cm (Fig. 5).

### Interrater and Intrarater Reliability

A total of fifty 3D images were annotated twice (ie, placement of the 3 required landmarks for registration), 1 week apart to avoid memory bias. Rater 1 had extensive experience with annotating and working with 3D images. Rater 2 had no relevant experience. Reliability was analyzed between the two raters to evaluate how subtle differences in the placement of the landmarks (nasion and both tragi) between and within raters affect the registration, and therefore, the automatically extracted measurements.

**FIGURE 6:**
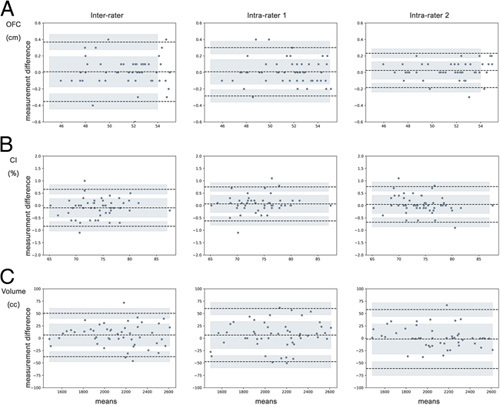
Blant-Altman plots for interrater and intrarater agreement of OFC (A), CI (B), and mesh volume above nasion-tragus plane (C). CI indicates Cephalic Index; OFC, occipitofrontal circumference.

Blant-Altman analysis was used to visualize the variation between and within raters (Fig. 6). Excellent intrarater and interrater reliability were found for both the OFC and CI (Supplemental Digital Content, Table 2, http://links.lww.com/SCS/F115). The MD and SE did not exceed 0.5 mm and 0.3 mm, respectively (interrater) with LoA ranging from −0.30 to 0.40 cm. Volumetric reliability was expressed as a ratio with respect to the mean volume (Supplemental Digital Content, Table 1, http://links.lww.com/SCS/F115) and showed a maximum mean error of 0.31% (interrater) and a maximum standard error of 0.19% (intrarater 2). Limits of agreement did not exceed 3%.

## DISCUSSION

The aim of this study was to compare the OFC measurements obtained using a tape measure versus those extracted automatically from 3D photogrammetry images (n = 264).

Based on the results from 264 measurements, a MD of −0.10 cm between the two methods was found, with LoA ranging from −0.93 cm to 0.74 cm, and a SE of 0.03 cm. The results showed an excellent agreement for practical purposes, but the question remains whether the 2 methods can be used interchangeably.

In practice, multiple professionals manually measure the OFC in accordance with the set protocol in our center and document the measurements within the electronic medical records. This means that subsequent OFC measurements are subject to either intrarater (when measured by the same professional) or interrater (when measured by a different professional) reliability problems. The potential error that is introduced when two different professionals manually measure the OFC is an important reference metric to determine whether the automated method from this study can be used interchangeably with manual methods.

Pastor-Pons and colleagues (2020) analyzed the interrater and intrarater reliability of manually measured cranial anthropometric measurements, including the OFC. Interrater reliability results of the manual OFC measurements showed a MD of −0.12 cm with a 95% CI ranging from −1.07 cm (−1.96 SD) to +0.84 cm (+1.96 SD). This means that the smallest detectable change or change beyond measurement error lies outside these LoA. The observed OFC LoA in our study when comparing manual to automated measurements (−0.93; 0.74) fall within these interrater manual LoA and can, therefore, be considered as a reliable alternative for manual measurements.

Because we propose an automated algorithm to extract the head measurements, the interrater and intrarater reliability can only be affected as a result of inaccurate or inconsistent annotation of the landmarks used for 3D image registration. One of the 2 raters had no experience with 3D imaging or digital annotation, to maximize the effect of this potential error.

Interrater and intrarater reliability results showed excellent agreement for the OFC and CI parameters with narrow LoA, indicating that the extraction algorithm is robust and not sensitive to small variations in landmark placements (Supplemental Digital Content, Table 2, http://links.lww.com/SCS/F115).

Very small mean volumetric differences (≤0.31%) and SEs (≤0.19%) were found, with slightly wider LoA compared with the OFC and CI measurements. This was expected since the position of the landmarks directly defines the nasion-tragus plane, along which the mesh is clipped, after which the volume above the plane is extracted. Because the extracted volume does not approximate the ICV and requires a correction based on CT/MRI (reducing the volume by 35%–45%), the absolute error is ultimately reduced by the same amount.

The field of computer vision is rapidly advancing, and it is now feasible to eliminate the manual annotation step. In most of our 3D imaging research nowadays, we use a fully automated nonrigid registration method that aligns two 3D meshes, enabling a completely automated pipeline that reduces potential reliability concerns arising from manual annotation.^30^ However, to use this method reliably, a 3D imaging protocol that standardizes patient orientation and position during image acquisition is essential. To ensure that our method can be used as an open-source tool, we chose a landmark-based registration approach, taking this prerequisite into account. As a result, users with hand-held 3D scanners or researchers who wish to apply our method to a preexisting data set without meeting this requirement can still fully leverage its potential.

It is worth emphasizing that various craniofacial centers use different types of devices and setups, which can result in differences in image quality. Therefore, the development of photogrammetry guidelines is essential for enhancing and standardizing image quality within and between centers. These guidelines should cover both image acquisition and preprocessing of data. As camera technology continues to evolve and improve, we anticipate that these guidelines, coupled with advancements in hardware and software, will lead to even greater improvements in measurement agreement for all segments of photogrammetry devices.

There are several limitations regarding the use of 3D photogrammetry for automated cranial measurements. First, these automated measurements are in some centers impossible because not every center possesses 3D photogrammetry equipment. Therefore, this solution is not available to everyone.

Of the 188 patients from whom a 3D image was acquired and included in this study, 85% are males (n = 160) and 15% are females (n = 28), which is higher than the reported 4:1 male predominance ratio seen in sagittal synostosis.^31,32^ The reason for the larger predominance of males in this study is due to the fact that more 3D images of female patients were excluded from the data collection process due to protruding hair.^27^ Long and protruding hair is an important limitation in craniofacial 3D photogrammetry when trying to analyze 3D images of older children. Strict imaging protocols regarding hairstyle and the use of a tight nylon hair cap can reduce the impact of long and protruding hair in craniofacial 3D photogrammetry. However, these limitations are less problematic in the pediatric population, where hair volume is typically not an issue, and automated measurements can increase reliability and reduce stress and discomfort caused by additional physical examinations. Despite the potential interference of hair on the automated measurements, we found that the correlation and measurement agreement remained consistent across the age spectrum and did not decrease with increasing OFC (ie, increasing age). This may be because manual measurements are similarly biased by hair.

Although manual measurement of the OFC using a tape measure is simple and reliable, it has the potential for human error and no option for retrospective assessment.^33–35^ Our results demonstrate that automated measurements using 3D images are a suitable alternative to manual measurements, without significant differences in reliability. Automated measurements from 3D photogrammetry also offer additional benefits, such as avoiding unnecessary stress in young patients and allowing for comprehensive retrospective analysis and comparison of the cranial morphology that extend well beyond the capability of traditional parameters.

The automated head measurement extraction algorithm presented in this study has been implemented in “CraniumPy,” an open-source framework for 3D image visualization, registration, and automated image optimization.^36^ A step-by-step guide on how to extract head measurements using CraniumPy can be found in the documentation on GitHub (https://github.com/T-AbdelAlim/CraniumPy/blob/master/resources/documentation.pdf).

## CONCLUSION

The results of this study indicate that the proposed algorithm for automated OFC measurements from 3D photogrammetry images is both robust and reliable, displaying a similar level of agreement to that observed in manual measurements. This method may be beneficial in young children who already undergo 3D imaging in craniofacial centers as part of their treatment protocol and in research settings that require a reproducible and transparent pipeline for anthropometric measurements. The primary benefit of using 3D photogrammetry lies in its ability to acquire high-dimensional data in a quick and safe manner and archive them for retrospective longitudinal analyses of the cranial morphology that can extend beyond these traditional parameters. Nevertheless, traditional parameters, such as the OFC, will remain a vital part of clinical practice and can be obtained in a quick and more reproducible manner from 3D photogrammetry data using the proposed method.

The algorithm for the automatic extraction of head measurements is available publicly through the open-source tool CraniumPy (https://github.com/T-AbdelAlim/CraniumPy).^36^ Further research is necessary to determine the optimal clinical workflow and cost-effectiveness of automated head measurements compared with manual measurements.

## Supplementary Material
