# Supplementary material for: Reliability and Agreement of Automated Head Measurements From 3-Dimensional Photogrammetry in Young Children
Source: J Craniofac Surg. 2023 Jun 12;34(6):1629–34. doi: 10.1097/SCS.0000000000009448 (PMC10445626; doi:10.1097/SCS.0000000000009448)
Supplement: Supplementary file 1 [file scs-34-1629-s001.pdf]

Supplementary Methods – Image Processing

Every 3D image or mesh that is going to be used for objective analysis needs to be registered in a reproducible manner. After data acquisition, we start with a source mesh (e.g. from a patient) in an arbitrary orientation that needs to be aligned with respect to a reference point or mesh (i.e. target), as shown in Figure 1. We use a normocephalic model of the head to visualize the alignment.[1]

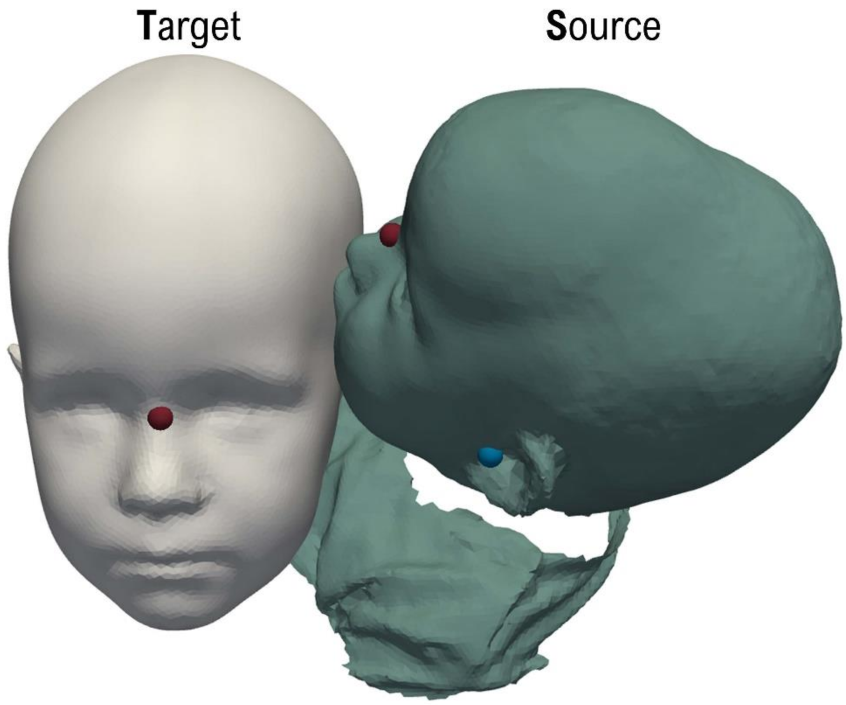

Figure 1: Target and source mesh before alignment

Three landmarks are used for the global alignment of a source mesh to the target mesh. Because the nasion and both tragi are relatively easy to identify on surface meshes of different resolutions, they serve as the primary landmarks in our registration pipeline. When the coordinates of these three landmarks are known, a triangular surface can be drawn between these vertices as shown in Figure 2. By simplifying the registration problem to the alignment of two triangles formed by the (user identified) landmark positions of the source

mesh and the known landmark positions of the target mesh, as visualized in Figure 3a, a quick global alignment of the source to the target can be achieved.

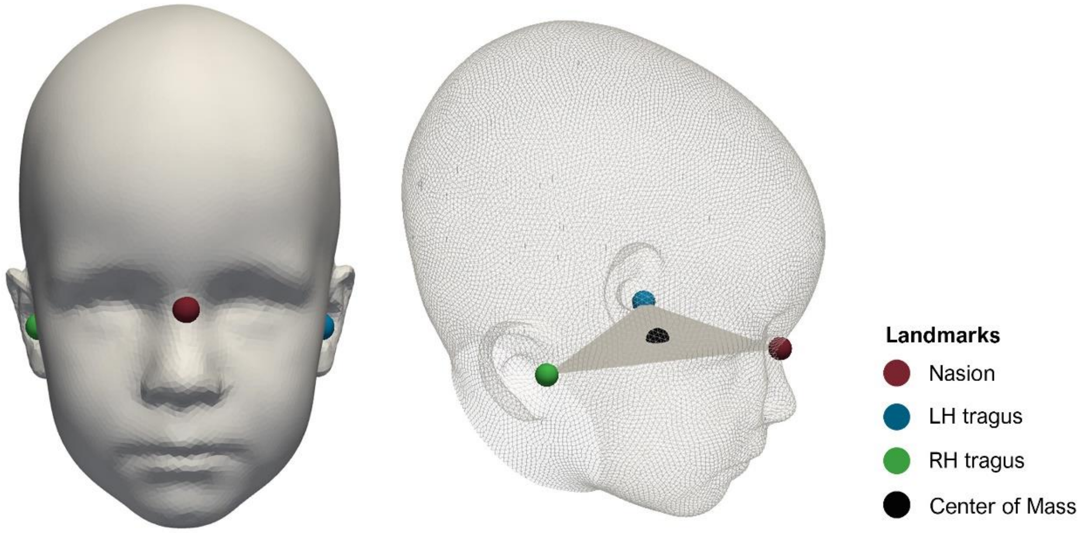

Figure 2: Registration using nasion, both tragi and the center of mass of the triangle formed by connecting the three landmarks

The centroid of the triangle in which the three vertices (N, L, R) correspond to the anatomical landmarks (Nasion, LH tragus, RH tragus) is obtained using *Eq. 1* and serves as the initial anchor point for the mesh.

$$C_s = \left(\frac{1}{3}(x_N + x_L + x_R), \frac{1}{3}(y_N + y_L + y_R), \frac{1}{3}\right) \qquad \textit{Eq. 1}$$

After the source mesh centroid (C_s) is calculated, the required translation to the target mesh is known as well, since the centroid of the target mesh (C_r) is defined as the origin $(0,0,0)$ of our reference frame. Therefore:

$$T_S = -C_S$$

With C_s located at the origin, the required rotations $R(\psi, \theta, \phi)$ are calculated and applied in that order (Figure 3b). *Eq. 2* is used calculate the angle θ between any two vectors $\boldsymbol{u}$ and $\boldsymbol{w}$.

The Rodrigues rotation formula (*Eq. 3*) shows that when rotating a vector $\boldsymbol{u}$ by θ radians around a unit axis $\boldsymbol{k}$, we obtain a new vector $\boldsymbol{u}'$.

$$\theta = arc\cos\frac{(\boldsymbol{u}\cdot\boldsymbol{w})}{(|\overline{\boldsymbol{u}}|\cdot|\overline{\boldsymbol{w}}|)} \qquad \textit{Eq. 2}$$

$$\boldsymbol{u}' = (1-cos(\theta))(\boldsymbol{u}\cdot\boldsymbol{k})\boldsymbol{k} + cos(\theta)\,\boldsymbol{u} + sin(\theta)\,\boldsymbol{k}\,x\,\boldsymbol{u} \qquad \textit{Eq. 3}$$

Rewritten in matrix notation:

$$\boldsymbol{u}' = \left((1-cos(\theta))\boldsymbol{K}^{\boldsymbol{2}} + I_3 + sin(\theta)\,\boldsymbol{K}\right)\boldsymbol{u} \qquad \textit{Eq. 4}$$

Where:

$$I_3 = \begin{bmatrix} 1 & 0 & 0 \\ 0 & 1 & 0 \\ 0 & 0 & 1 \end{bmatrix}$$

$$K = \begin{bmatrix} 0 & k_z & -k_y \\ -k_z & 0 & k_x \\ k_y & -k_x & 0 \end{bmatrix}$$

The term $\left((1-\cos(\theta))\boldsymbol{K}^{\boldsymbol{2}} + I_3 + \sin(\theta)\,\boldsymbol{K}\right)$ in *eq. 4* is known as the rotation matrix R that is used to perform the desired rotation. Every vertex of a mesh can be seen as a vector from a defined origin and therefore, multiplying every vertex of a mesh with the found rotation matrix will result in a rotation of the entire mesh around this origin.

Because rotations of vectors in three dimensions are not commutative, the order in which we apply the sequence of rotations is important. For our application, we apply the rotations in the order $R_zR_yR_x$. In the example presented in Figure 3b, this looks as follows:

1. Rz rotates the mesh psi radians in counter-clockwise (+) direction to align the two nasion vectors ($\hat{\boldsymbol{u}}_S$ and $\hat{\boldsymbol{u}}_T$) in the xy-plane.

z-axis sign convention: inferior (-) to superior (+)

2. Ry rotates the mesh theta radians in counter-clockwise (+) direction to align the two normal vectors ($\hat{\boldsymbol{n}}_S$ and $\hat{\boldsymbol{n}}_T$) in the xz-plane.

y-axis sign convention: posterior (-) to anterior (+)

3. Rx finally tilts the mesh phi radians in clockwise (-) direction to align the two normal vectors ($\hat{\boldsymbol{n}}_S$ and $\hat{\boldsymbol{n}}_T$) in the yz-plane.

x-axis sign convention: left (-) to right (+)

Note that in the latest versions of CraniumPy (v.0.3.0 and above), we have introduced an additional rotation of the registered mesh based on the more commonly used right hand coordinate system in which the z-axis points outward from the nose.

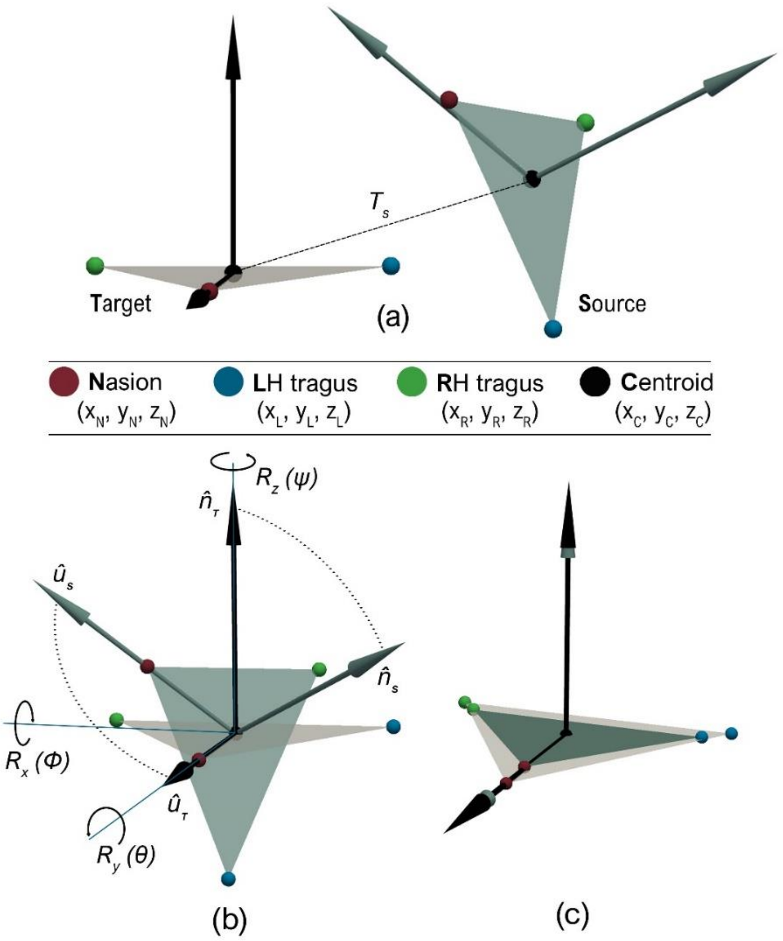

Figure 3: Registration steps: (a) translation from source to target, (b) rotations along the three axes to align the two vectors, (c) result after registration

Now that the mesh is aligned in a standardized manner, head measurements can be automatically extracted as described in the methods section of the paper. However, to increase the reproducibility and accuracy of the automated measurements, a final step is implemented that reduces the size of the mesh, removes any potential artifacts, and harmonizes the resolution of the output data (default resampling: $n_{vertices} = 10.000$)

With the location of the nation and both tragi known, each mesh is clipped along this plane to reduce the mesh such that it's volume can be correlated to the intracranial volume (e.g. based on CT or MRI data). This reduced mesh is then uniformly resampled using a python implementation of the conventional Approximated Centroidal Voronoi Diagrams (ACVD) algorithm, which is a memory efficient technique for uniform tessellation of polygonal meshes.[2,3]

Movement induced artifacts or any other errors in the mesh reconstruction can compromise analysis results. It is therefore important to convert every mesh into a single manifold (watertight) structure that does not contain intersecting or degenerate mesh elements. To convert each cranial mesh into such a structure, we make use of PyMeshFix, a python implementation of the (C++) MeshFix software.[4] This implementation makes sure that all singularities, self-intersections and degenerate elements are removed from any cranial mesh, without affecting artifact-free regions.

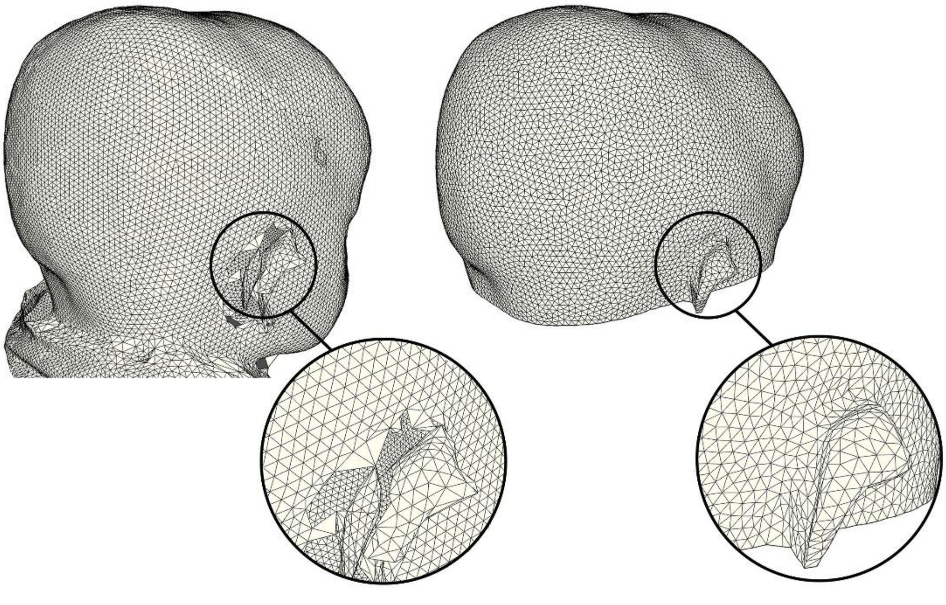

Bibliography

1. T. Huysmans, L. Goto, J. Molenbroek RG. DINED Mannequin. Tijdschr voor Hum Factors. 2020;45(1):4--7.
2. pyvista/pyacvd: Python implementation of surface mesh resampling algorithm ACVD [code]. Available from: https://github.com/pyvista/pyacvd
3. Valette S, Chassery JM. Approximated Centroidal Voronoi Diagrams for Uniform Polygonal Mesh Coarsening. Comput Graph Forum. 2004;23(3 SPEC. ISS.):381–9.
4. Attene M. A lightweight approach to repairing digitized polygon meshes. Vis Comput. 2010;26(11):1393–406.
