# Supplementary material for: Reliability and Agreement of Automated Head Measurements From 3-Dimensional Photogrammetry in Young Children
Source: J Craniofac Surg. 2023 Jun 12;34(6):1629–34. doi: 10.1097/SCS.0000000000009448 (PMC10445626; doi:10.1097/SCS.0000000000009448)
Supplement: Supplementary file 2 [file scs-34-1629-s002.docx]

| Measurements from 3D images (n=50) | Mean ± SD |
| --- | --- |
| Mean age (months) | 41.8 ± 21.5 |
| Mean OFC (cm) | 51.36 ± 2.46 |
| Mean CI (%) | 74.18 ± 4.15 |
| Mean Mesh Volume* (cc) | 2104.25 ± 293.98 |
| *mesh volume above nasion-tragus plane, not corrected to approximate intracranial volume | |

*Supplemental Table 1: Mean measurement values from 3D image subset (n=50) for inter-rater and intra-rater reliability*

|  | Inter-rater | Intra-rater 1 | Intra-rater 2 |
| --- | --- | --- | --- |
|  | OFC from 3D (cm) – absolute | | |
| Mean difference | 0.08 ± 0.18 | 0.01 ± 0.15 | 0.02 ± 0.11 |
| Limits of agreement | [-0.35 ; 0.37] | [-0.29 ; 0.30] | [-0.18 ; 0.23] |
| Standard error | 0.03 ± 0.05 | 0.02 ± 0.04 | 0.02 ± 0.03 |
|  | CI from 3D (%) – absolute | | |
| Mean difference | -0.009 ± 0.38 | 0.064 ± 0.35 | 0.04 ± 0.37 |
| Limits of agreement | [-0.84 ; 0.65] | [-0.62 ; 0.75] | [-0.68 ; 0.77] |
| Standard error | 0.06 ± 0.10 | 0.05 ± 0.09 | 0.05 ± 0.09 |
|  | Volume from 3D (%) – relative | | |
| Mean difference | 0.31 ± 1.06 | 0.30 ± 1.30 | -0.08 ± 1.44 |
| Limits of agreement | [-1.77 ; 2.39] | [-2.25 ; 2.85] | [-2.91 ; 2.75] |
| Standard error | 0.14 ± 0.24 | 0.17 ± 0.29 | 0.19 ± 0.33 |

Supplemental Table 2: Inter-rater and intra-rater reliability of measurements (OFC, CI, Volume), automatically extracted from a subset of 3D images (n=50)
